# Unhealthy behaviours and risk of visual impairment: The CONSTANCES population-based cohort

**DOI:** 10.1038/s41598-018-24822-0

**Published:** 2018-04-26

**Authors:** Bénédicte M. J. Merle, Gwendoline Moreau, Anna Ozguler, Bernard Srour, Audrey Cougnard-Grégoire, Marcel Goldberg, Marie Zins, Cécile Delcourt

**Affiliations:** 1 University of Bordeaux, Inserm, Bordeaux Population Health Research Center, team LEHA, UMR 1219, F-33000 Bordeaux, France; 2 UMS 11, Cohortes épidémiologiques en population, Inserm-UVSQ, Villejuif, France; 3 UMS 11, Cohortes épidémiologiques en population, Inserm-UVSQ, Paris Descartes University, Villejuif, France

## Abstract

Unhealthy behaviours are linked to a higher risk of eye diseases, but their combined effect on visual function is unknown. We aimed to examine the individual and combined associations of diet, physical activity, smoking and alcohol consumption with visual impairment among French adults. 38 903 participants aged 18–73 years from the CONSTANCES nationwide cohort (2012–2016) with visual acuity measured and who completed, lifestyle, medical and food frequency questionnaires were included. Visual impairment was defined as a presenting visual acuity <20/40 in the better eye. After full multivariate adjustment, the odds for visual impairment increased with decreasing diet quality (p for trend = 0.04), decreasing physical activity (p for trend = 0.02) and increasing smoking pack-years (p for trend = 0.03), whereas no statistically significant association with alcohol consumption was found. Combination of several unhealthy behaviours was associated with increasing odds for visual impairment (p for trend = 0.0002), with a fully-adjusted odds ratio of 1.81 (95% CI 1.18 to 2.79) for participants reporting 2 unhealthy behaviours and 2.92 (95% CI 1.60 to 5.32) for those reporting 3 unhealthy behaviours. An unhealthy lifestyle including low/intermediate diet quality, low physical activity and heavy smoking was associated with visual impairment in this large population-based study.

## Introduction

Visual impairment is estimated to affect 191 million people and 33 million are thought to be blind worldwide^1^. People with impaired vision experience a reduced quality of life^2,3^, greater difficulty in their daily lives and social dependence^4,5^. Vision loss is also associated with adverse health outcomes such as depression^6,7^, falls and fractures^8^, leading to a considerable burden for the individual and the family, as well as higher health care costs for society. Uncorrected refractive errors, age-related eye diseases (cataract, age-related macular degeneration (AMD), glaucoma and diabetic retinopathy) are the major causes of visual impairment in adults worldwide^9^. Since 1999, prevention of visual impairment and blindness has been a priority of the World Health Organization^10,11^. While many efforts have been developed in secondary and tertiary prevention of visual impairment, there is a need to better characterize the potential for primary prevention of visual impairment.

Unhealthy behaviours, such as low diet quality, low physical activity, smoking and heavy drinking are modifiable factors that may contribute to the primary prevention of visual impairment. Indeed, in the past 20 years, epidemiological studies have highlighted that low dietary intake of antioxidants and omega-3 fatty acids^12–20^, low physical activity^21–23^ and smoking^12,24–26^ were associated with an increased risk of eye diseases. Associations with alcohol consumption are less clearly defined^27–33^. However, very few studies have examined the global impact of unhealthy behaviours on vision. Moreover, people have a propensity to follow common behavioural patterns, and unhealthy behaviours, often clustered, may have synergistic effects on health^34,35^, underlining the importance to examine their combined effects. There is evidence that the risk of coronary disease^36^, cardiovascular events^37^, cancer^38^, diabetes^39^, poor cognitive function^40^ and mortality^41,42^ increase with the number of unhealthy behaviours. The few studies that have examined the combined effect of these unhealthy behaviours on ocular health were focused on AMD^43–45^. However, to our knowledge, no study has examined the combined effect of unhealthy behaviours on visual function.

The French nationwide, large CONSTANCES cohort^46^ represents a major opportunity for a better knowledge of the epidemiology of visual impairment. Our objective is to examine the individual and combined associations of diet, physical activity, smoking and alcohol consumption with visual impairment in the CONSTANCES cohort.

## Results

### Characteristics of participants

As shown in Table 1, among 38 903 participants, 228 (0.59%) were visually impaired. Visual impairment was significantly more frequent in older participants (p < 0.0001) and was slightly more frequent in women, although non-significant (p = 0.09). After adjustment for age and sex, it was significantly more frequent in participants with lower education (p = 0.005), lower monthly income (p < 0.0001), but was not significantly associated with diabetes (p = 0.26), hypertension (p = 0.38), hypercholesterolemia (p = 0.49), body mass index (BMI) (p = 0.31) and alcohol consumption (p = 0.09).

**Table 1:** Frequency of visual impairment according to characteristics of participants (n = 38 903).

Characteristics	Number of participants	Visual impairment n (%)	Age and sex adjusted P value^a^
Overall	38 903	228 (0.59)	
**Sociodemographic characteristics**			
Age, year			<*0*.*0001*
[18–30]	5 953	13 (0.22)	
[30–40]	7 452	16 (0.21)	
[40–50]	8 820	26 (0.29)	
[50–60]	8 513	59 (0.69)	
[60–70]	7 833	107 (1.37)	
≥70	332	7 (2.11)	
Sex			*0*.*09*
Male	18 333	99 (0.54)	
Female	20 570	129 (0.63)	
Educational level			*0*.*005*
≤Primary school	3 080	37 (1.20)	
Secondary school	5 783	46 (0.80)	
High school	6 902	41 (0.59)	
≤Bachelor level	10 165	46 (0.45)	
≥Master level or equivalent	12 973	58 (0.45)	
Monthly Income, euros/household			<*0*.*0001*
<1500	4 205	65 (1.55)	
1500–2800	10 291	60 (0.58)	
2800–4200	11 395	50 (0.44)	
≥4200	10 902	43 (0.39)	
No answer	2 110	10 (0.47)	
**Related diseases**			
Diabetes			*0*.*26*
No	37 293	212 (0.57)	
Yes	1 610	16 (0.99)	
Hypertension			*0*.*38*
No	28 627	137 (0.48)	
Yes	10 276	91 (0.89)	
Hypercholesterolemia			*0*.*49*
No	28 048	134 (0.48)	
Yes	10 855	94 (0.87)	
Body mass index, kg/m²			*0*.*31*
<25	22 734	112 (0.49)	
25–30	11 656	85 (0.73)	
≥30	4 513	31 (0.69)	
Alcohol consumption			*0*.*09*
Never or light drinkers	14 378	86 (0.60)	
Moderate drinkers	18 526	98 (0.51)	
Heavy drinkers	4 676	36 (0.90)	
No answer	1 323	8 (0.60)	

^a^*p* from mixed logistic regression model adjusted for age and sex with random intercept for inclusion center.

### Visual impairment, diet quality, physical activity, smoking and unhealthy behaviours

**Figure 1:**
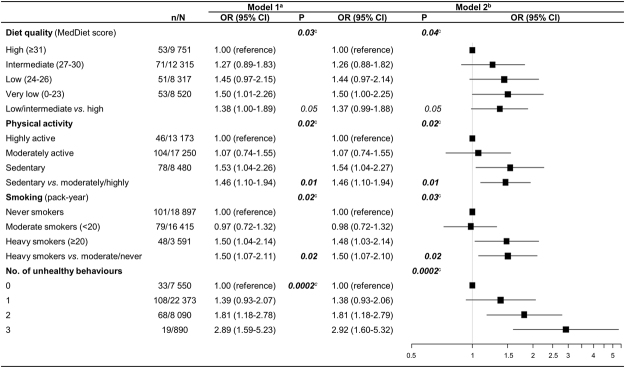
Associations of visual impairment with diet quality, physical activity, smoking and unhealthy behaviours (n = 38 903). (**a**) Mixed logistic regression model adjusted for age, sex, education level and monthly income with random intercept for inclusion center. (**b**) Mixed logistic regression model adjusted for age, sex, education level, monthly income, diabetes, hypertension, hypercholesterolemia and body mass index with random intercept for inclusion centre. (**c**) p for trend. CI: confidence interval, MedDiet: Mediterranean Diet; OR: odds ratio.

Figure 1 reports associations of visual impairment with diet quality, physical activity, smoking status and the number of unhealthy behaviours. After adjustment for age, sex, education and monthly income (Model 1), visual impairment was significantly associated with diet quality (p = 0.03), physical activity (p = 0.02), smoking status (p = 0.02) and the number of unhealthy behaviours (p = 0.0002). After further adjustment for characteristics significantly associated with the number of unhealthy behaviours (diabetes, hypertension, hypercholesterolemia and BMI (Model 2), these associations remained significant.

Participants reporting a low/intermediate diet quality had a 1.37-fold increase (odd ratio 1.37 95% confidence interval 0.99–1.88) of the odds of visual impairment, this association did not reach statistical significance when a binary variable was used for diet. Sedentary participants had a 1.46 (1.10–1.94)-fold increase of the odds of visual impairment compared to moderately/highly active participants. Heavy smokers had a 1.50 (1.07–2.10)-fold increase of the odds of visual impairment compared with never/moderate smokers. The frequency of visual impairment increased with the number of unhealthy behaviours (p-trend = 0.0002). Participants reporting two or three unhealthy behaviours had a 1.81 to 2.92-fold increase of the odds of visual impairment, respectively. After additional adjustment for alcohol, odds ratio for diet quality, physical activity, smoking and unhealthy behaviours remained unchanged consumption (data not shown).

Table 2 displays participants’ characteristics according to the number of unhealthy behaviours. Approximatively 58% of participants had one unhealthy behaviour, 21% had two, 2% had three and 19% had none. Unhealthy behaviours were more frequent in older participants (p < 0.0001) and in men (p < 0.0001). After adjustment for age and sex, a higher number of unhealthy behaviours was associated with lower education, lower monthly income, presence of diabetes, hypertension and hypercholesterolemia, higher BMI and heavy drinking (all p < 0.0001). This table also describes diet quality, physical activity and smoking according to the number of unhealthy behaviours.

**Table 2:** Characteristics of participants according to the number of unhealthy behaviours (n = 38 903).

Characteristics	Number of unhealthy behaviours				P value age and sex adjusted^a^
	0	1	2	3	
N (%) overall	7 550 (19.41)	22 373 (57.51)	8 090 (20.80)	890 (2.29)	
**Sociodemographic characteristics**					
Age, year					<*0*.*0001*
[18–30]	850 (11.26)	3 636 (16.25)	1 466 (18.12)	1 (0.11)	
[30–40]	1 275 (16.89)	4 942 (22.09)	1 201 (14.85)	34 (3.82)	
[40–50]	1 745 (23.11)	5 388 (24.08)	1 541 (19.05)	146 (16.40)	
[50–60]	1 930 (25.56)	4 565 (20.40)	1 737 (21.47)	281 (31.57)	
[60–70]	1 693 (22.42)	3 669 (16.40)	2 062 (25.49)	409 (45.96)	
≥70	57 (0.75)	173 (0.77)	83 (1.03)	19 (2.13)	
Sex					<*0*.*0001*
Male	2 361 (31.27)	11 041 (49.35)	4 304 (53.20)	627 (70.45)	
Female	5 189 (68.73)	11 332 (50.65)	3 786 (46.80)	263 (29.55)	
Education					<*0*.*0001*
≤Primary school	441 (5.84)	1 588 (7.10)	888 (10.98)	163 (18.31)	
Secondary school	846 (11.21)	3 313 (14.81)	1 404 (17.35)	220 (24.72)	
High School	1 179 (15.62)	3 909 (17.47)	1 662 (20.54)	152 (17.08)	
≤Bachelor level	2 071 (27.43)	6 035 (26.97)	1 883 (23.28)	176 (19.78)	
≥Master level or equivalent	3 013 (39.91)	7 528 (33.65)	2 253 (27.85)	179 (20.11)	
Monthly Income, euros/household					<*0*.*0001*
<1500	667 (8.83)	2 136 (9.55)	1 244 (15.38)	158 (17.75)	
1500–2800	1 859 (24.62)	5 887 (26.31)	2 277 (28.15)	268 (30.11)	
2800–4200	2 216 (29.35)	6 874 (30.72)	2 071 (25.60)	234 (26.29)	
≥4200	2 437 (32.28)	6 335 (28.32)	1 944 (24.03)	186 (20.90)	
No answer	371 (4.91)	1 141 (5.10)	554 (6.85)	44 (4.94)	
**Related diseases**					
Diabetes					<*0*.*0001*
No	7 341 (97.23)	21 579 (96.45)	7 601 (93.96)	772 (86.74)	
Yes	209 (2.77)	794 (3.55)	489 (6.04)	118 (13.26)	
Hypertension					<*0*.*0001*
No	5 841 (77.36)	16 894 (75.51)	5 464 (67.54)	428 (48.09)	
Yes	1 709 (22.64)	5 479 (24.49)	2 626 (32.46)	462 (51.91)	
Hypercholesterolemia					<*0*.*0001*
No	5 429 (71.91)	16 679 (74.55)	5 499 (67.97)	441 (49.55)	
Yes	2 121 (28.09)	5 694 (25.45)	2 591 (32.03)	449 (50.45)	
Body mass index, kg/m²					<*0*.*0001*
<25	5 213 (69.05)	13 258 (59.26)	3 961 (48.96)	302 (33.93)	
25–30	1 805 (23.91)	6 805 (30.42)	2 704 (33.42)	342 (38.43)	
≥30	532 (7.05)	2 310 (10.32)	1 425 (17.61)	246 (27.64)	
Alcohol consumption					<*0*.*0001*
Never or light drinkers	3 693 (48.91)	7 692 (34.38)	2 783 (34.40)	210 (23.60)	
Moderate drinkers	3 354 (44.42)	11 718 (52.38)	3 726 (46.06)	386 (43.37)	
Heavy drinkers	269 (3.56)	2 312 (10.33)	1 192 (14.73)	245 (27.53)	
No answer	234 (3.10)	651 (2.91)	389 (4.81)	49 (5.51)	
MedDiet score					
High (≥31)	7 550 (100.00)	2 074 (9.27)	127 (1.57)	0 (0.00)	
I ntermediate (27–30)	0 (0.00)	9 133 (40.82)	2 938 (36.32)	244 (27.42)	
Low (24–26)	0 (0.00)	5 778 (25.83)	2 295 (28.37)	244 (27.42)	
Very low (0–23)	0 (0.00)	5 388 (24.08)	2 730 (33.75)	402 (45.17)	
Physical activity					
Highly active	3 291 (43.59)	9 146 (40.88)	736 (9.10)	0 (0.00)	
Moderately active	4 259 (56.41)	11 636 (52.01)	1 355 (16.75)	0 (0.00)	
Sedentary	0 (0.00)	1 591 (7.11)	5 999 (74.15)	890 (100.00)	
Smoking (pack-year)					
Never smokers	4 428 (58.65)	11 316 (50.58)	3 153 (38.97)	0 (0.00)	
Moderate smokers (<20)	3 122 (41.35)	10 574 (47.26)	2 719 (33.61)	0 (0.00)	
Heavy smokers (≥20)	0 (0.00)	483 (2.16)	2 218 (27.42)	890 (100.00)	

^a^*p* from mixed multinomial logistic regression model adjusted for age and sex with random intercept for inclusion center. Values are number of individuals (%).

### Sensitivity analyses

Associations between the number of unhealthy behaviours and visual impairment did not differ when using a different cut-off for diet quality (low/very low versus intermediate/high, Table 3). Participants reporting one, two or three unhealthy behaviours had a 1.17 (0.85–1.61), 1.89 (1.31–2.72) and 2.14 (1.12–4.09)-fold increase of the odds of visual impairment, respectively. Associations were statistically significant for reporting two or three unhealthy beaviours. Besides, we performed multiple imputations for missing data, in order to assess a potential selection bias. The associations between the number of unhealthy behaviours and visual impairment remained unchanged. Participants reporting one, two or three unhealthy behaviours had a 1.21 (0.87–1.69), 1.47 (1.03–2.10) and 2.37 (1.49–3.79)-fold increase of the odds of visual impairment, respectively.

**Table 3:** Associations of visual impairment with the number of unhealthy behaviours using different cut-off and imputed data. Sensitivity analyses.

No. of unhealthy behaviours	N	Model 1^a^		Model 2^b^	
		OR (95% CI)	P	OR (95% CI)	P
MedDiet score (low/very low vs. intermediate/high)	38 903		*0*.*0003*		*0*.*0004*
0		1.00 (reference)		1.00 (reference)	
1		1.17 (0.86–1.61)		1.17 (0.85–1.61)	
2		1.88 (1.31–2.70)		1.89 (1.31–2.72)	
3		2.12 (1.12–4.03)		2.14 (1.12–4.09)	
Multiple imputations for missing data	52 035		<*0*.*0001*^*c*^		*0*.*0003*^*c*^
0		1.00 (reference)		1.00 (reference)	
1		1.23 (0.89–1.70)		1.21 (0.87–1.69)	
2		1.53 (1.08–2.17)		1.47 (1.03–2.10)	
3		2.52 (1.59–4.00)		2.37 (1.49–3.79)	

^a^Mixed logistic regression model adjusted for age, sex, education level and monthly income with random intercept for inclusion center.

^b^Mixed logistic regression model adjusted for age, sex, education level, monthly income, diabetes, hypertension, hypercholesterolemia and body mass index with random intercept for inclusion center.

^c^p for trend.

## Discussion

Modifiable unhealthy behaviours, such as low diet quality, sedentary behaviour and heavy smoking, were associated with increased odds for visual impairment in this large French study, after adjustment for sociodemographic characteristics and cardiovascular risk factors. The odds of visual impairment increased with the number of unhealthy behaviours. Participants with three unhealthy behaviours had a 2.9-fold increased odds of visual impairment compared to those without unhealthy behaviours.

Consistent with our results, the Beaver Dam Eye Study (BDES), a prospective cohort study, showed that sedentary behaviour was associated with an increased risk for visual impairment^47^. In the BDES, association with smoking was not significant while current smoking was associated with a higher rate of self-reported visual impairment among participants aged 50 years or more with age-related eye diseases in the Behavioral Risk Factor Surveillance System (BRFSS)^48^, a cross-sectional telephone survey.

Regarding alcohol consumption, the BDES suggested that alcohol consumption in the past was associated with an increased risk for visual impairment^47^. This association was also found among participants included in the BRFSS. Fan *et al*. reported that consuming more than one drink per day and binge drinking were both associated with self-reported visual impairment^33^. Our study did not report significant association between alcohol consumption and visual impairment. To our knowledge, our study is the only one to assess association between diet quality and visual impairment.

AMD, cataract, glaucoma and diabetic retinopathy, are major causes of visual impairment and therefore associations between unhealthy behaviours and visual impairment could be explained by the association between these eye diseases and unhealthy behaviours. A low diet quality, including low intake of antioxidants and omega3 fatty acids as well as a low adherence to the Mediterranean-type diet, is associated with a higher risk of AMD^12–17,19^, diabetic retinopathy^18,20^ and cataract^24,49,50^. Smoking is a well-known risk factor for AMD^12,26^ and cataract^24,25^ and has been associated with an increased risk for glaucoma^51,52^ and uveitis^53^. Low physical activity has been associated to a higher risk for AMD^12,23,44^. Associations of eye diseases with alcohol consumption are inconsistent across studies^27–33^.

Most studies on visual impairment and eye diseases have examined behaviours separately and not their cumulative effect. People have a propensity to follow common behavioural patterns and unhealthy behaviours, often clustered, may have synergistic health effects, underlining the importance of examining their combined effects^34,35^. Mares *et al*. reported the combined effect of diet, physical activity and smoking on the risk of AMD in US women aged 55–74^44^. In this study, a high diet quality and a high level of physical activity were associated with a lower prevalence of AMD. Although smoking was not associated with AMD, having a combination of these three healthy behaviours was associated with lower odds for AMD. Consistently with our study, this study showed that behaviours had cumulative effects and that the risk of AMD decreased progressively with the healthy behaviours. Recently, Gopinath *et al*. reported that the odds of AMD rose as the number of unhealthy behaviours (smoking, alcohol, low physical activity and diet) increased, in participants aged 49+ in the Blue Mountains Eye Study^45^.

The cross-sectional design of the present study represents a limitation. In cross-sectional analyses, reverse causation cannot be excluded. Participants with visual impairment might have changed their behaviours, especially diet and physical activity due to vision loss and people who are less health conscious may have a lower presenting visual acuity due to lower utilization of eye care services, or other behavioural or environmental factors that are associated with inadequate diet, decreased exercise or smoking. CONSTANCES is a prospective cohort study, so we expect to have prospective data in the future. Another limitation of this study is related to missing data and selection bias. We therefore used multiple imputations in order to assess the potential selection bias due to missing data. In the imputed dataset, the associations between the number of unhealthy behaviours and visual impairment remained in the same range. In addition, participants in the CONSTANCES cohort might be more health conscious and have a healthier lifestyle and better ocular health than non-participants. This may have affected the frequency of visual impairment and frequency of unhealthy behaviours. However, data collection was performed in the same way in all participants. We can assume that the error was not differential and was unlikely to have biased the estimation of the associations of visual impairment with unhealthy behaviours.

In our study, physical activity was assessed using a score which is less reproducible than using metabolic equivalent of task and does not allow us to compare physical activity levels with other studies. Nevertheless, physical activity was estimated using physical activity at work and outside work which is a better evaluation of total physical activity than leisure/sport activity only.

Our results suggest a linear effect of diet on visual function and using a dichotomous variable was questionable. To assess whether associations between the number of unhealthy behaviours and visual impairment may differ using a different cut-off for diet quality, we used the median value of the MedDiet score as an alternate cut-off and the results remained unchanged.

This study’s main strengths include a randomly selected and large nationwide sample providing high statistical power. All data were recorded using standardized examination and questionnaires. Compared to survey studies^33,48^, our study has the advantage of having presenting visual acuity measured according to the international standards rather than self-reported visual impairment. Another major strength of our study is the exploration of the combined effect of unhealthy behaviours.

Our results are consistent with studies on coronary disease^36^, cardiovascular events^37^, cancer^38^, diabetes^39^, disability^54^, poor cognitive function^40^, mortality^41,42^ and AMD^44^ showing unhealthy behaviours have cumulative effects. For the last several years, diet quality, physical activity, smoking and heavy drinking have been targeted by public health policies by many countries, and some of them such as smoking, have shown decreasing trends^55^. Maintaining and developing primary prevention and public health policy to encourage healthier lifestyles could lead to decreasing future trends in visual impairment. A review recently showed that the age-standardized prevalence of visual impairment has decreased in the past 20 years^1^. This trend might partially be explained by decreased trends in smoking and other unhealthy behaviours, together with progress in eye care. Thus, the present study adds an argument towards targeting the potential benefit of multi-behaviours interventions to reduce the burden of visual impairment and improve ocular health.

This study suggests that an unhealthy lifestyle, characterized by a low diet quality, sedentary behaviour and heavy smoking, is associated with greater odds of visual impairment, which increased with the number of these unhealthy behaviours. These findings are of utmost importance for primary prevention, as these behaviors are modifiable and interventions aimed at promoting a global healthy lifestyle may help improving ocular health.

## Methods

### Study population

The CONSTANCES cohort is a prospective cohort study of general adult population randomly selected among the French National Health Insurance Fund database^46^ (http://www.constances.fr/index_EN.php). A total of 200 000 participants are expected to be included over a 6-year period (2012–2018).

At inclusion, the randomly selected subjects are invited to attend one of the 21 selected health screening centers^46^ for a comprehensive health examination. During the examination, weight, height, blood pressure and vision were measured by trained nurses and laboratory tests were performed according to standardized operational procedures, included in an extensive quality control program^46,56^. Health events, including diabetes, hypercholesterolemia and hypertension, were recorded by a physician during the medical examination. Sociodemographic (age, sex, education and monthly income), health events and behaviours (diet, physical activity, smoking and alcohol consumption) were collected using a self-administrated questionnaire completed at home.

All the participants included in the CONSTANCES cohort have signed an informed consent form. This research follows the tenets of the Declaration of Helsinki and was approved by the National Data Protection Authority (*Commission Nationale Informatique et Libertés*) and the Institutional Review Board of the National Institute for Medical Research and the local Committee for Persons Protection (*Comité de Protection des Personnes*).

The study reported here is based on available data in 2017 collected from February 2012 to January 2016 and age range for participants was 18 to 73 years.

### Visual impairment

Presenting distance visual acuity (using current refractive correction, if any) was measured in each eye using the Snellen scale according to a standard operating procedure, by trained nurses in each health screening center at inclusion. Visual impairment was defined as a presenting visual acuity <20/40 in the better eye, as in other studies^57^.

### Unhealthy behaviours

#### Diet assessment

Data on food consumption were collected at enrolment with a validated self-administered 40-items food frequency questionnaire (FFQ). Subjects were asked to report how often they consumed each food or beverage item. Consumption was classified into 6 categories from “never or almost never” to “one serving per day or more, if more than once a day indicate the number of serving per day”.

**Figure 2:**
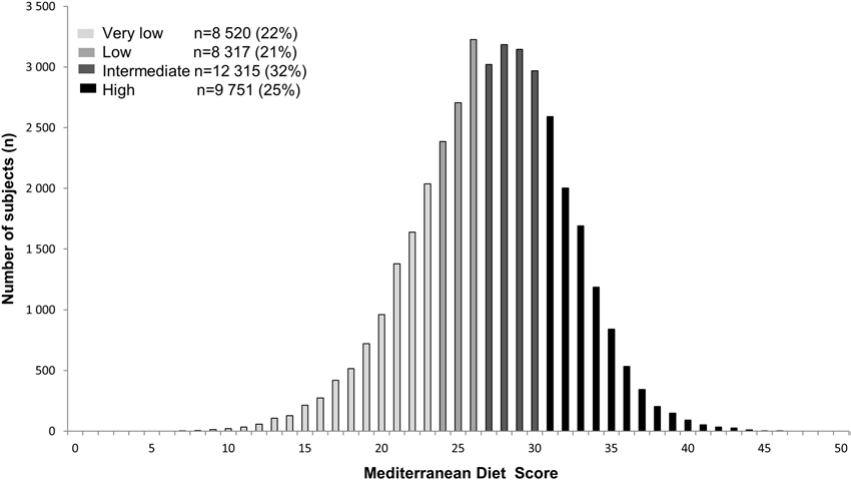
Description of the Mediterranean diet score (MedDiet) (n = 38 903).

To assess diet quality, we used the MedDiet score developed by Panagiotakos *et al*.^58^. According to our FFQ, we made a MedDiet score with 10 food groups: cereals (refined and non-refined), fruits, vegetables, legumes, fish, red meat and products, poultry, dairy products, olive oil, alcoholic beverages. The weekly intake of each food or beverage group was calculated as the sum of the number of serving consumed per week. For each food group hypothesized to benefit health (cereals, fruits, vegetables, legumes, fish) 0 to 5 points were given according to the number of serving/week: 0 point for non-consumers, 1 point for [0–1], 2 points for [1–2], 3 points for [2–3], 4 points for [3–4.5] and 5 points for a consumption over than 4.5 servings/week. For components presumed to be detrimental to health (read meat, poultry, dairy products) 0 to 5 points as follow: 0 for a consumption over than 4.5, 1 point for [3–4.5], 2 points for [2–3], 3 points for [1–2], 4 points for [0–1] and 5 points for non-consumers. For olive oil use 0 point was given for non-users, 1 point for rare, 2 for less than 0.25, 3 point for [0.25–0.75], 4 points for [0.75–7] servings/week and 5 point for a daily use. For alcohol consumption 5 points were given for a consumption more than 0 glass/week and less than 3 glass/week, 4 points for [3–4], 3 points for [4–5], 2 points for [5–6], 1 point for [6–7] and 0 for >7 glass/week or 0 glass/week. For each participant, the total MedDiet score was calculated by adding the scores (0 to 5 points) for each food group. Scores ranged from 0 (low diet quality) to 50 (high diet quality). According to the quartiles of distribution of the MedDiet score, we defined diet quality as very low (0–23), low (24–26), intermediate (27–30) and high (≥31) (Fig. 2). We considered very low/low/intermediate scores to be an unhealthy behaviour.

#### Physical activity

Physical activity at work was evaluated for participants who currently work or had worked in the past, excluding those who had never worked. Subjects were asked to evaluate what kind of physical effort they usually did in their current job for current workers or in their last job for past workers. A physical activity score at work was created: 1 point for “sedentary”, 2 for “moderately active” and 3 for “highly active” subjects.

Physical activity outside work was assessed through questions on frequency of regular trips (walking, biking…), sports (running, football, tennis…) and leisure activities (gardening, cleaning house…) during the past year. For each of those 3 variables we used a 2-points scale as follow: for regular trip, 2 points were given for answering “Yes, 15 minutes or more/trip”, 1 point for “Yes, less than 15 minutes/trip” and 0 point for “No”. For sports and leisure activities, 2 points were given for answering “Yes, 2 hours or more/week”, 1 point for “Yes, less than 2 hours /week” and 0 point for “No”. Then the physical activity outside work was summed from 0 to 6 and a physical activity score outside work was created: 1 point “sedentary” (0–1), 2 “moderately active” (2–3) and 3 “highly active (4–6 highly active).

To compute a unique physical activity variable accounting for physical activity at work and outside work, we added the two indicators detailed above and classified subjects according to 3 physical activity levels: “sedentary (score 1–2)”, “moderately active” (score 3) and “highly active” (score 4–6). We considered the “sedentary” level to be an unhealthy behaviour.

#### Smoking status

Smoking status was assessed using a self-administrated questionnaire and the number of pack-years (PY) (PY = packs (20 cigarettes) smoked per day X years of smoking) was calculated for each current or former smoker. Smoking status was defined as follow: never smoker, moderate smokers (<20 PY) and heavy smokers (≥20 PY)^59^. We considered heavy smoking to be an unhealthy behaviour.

Alcohol consumption: Data on alcohol consumption were collected at enrolment with a validated self-administered questionnaire. Alcohol consumption was defined as never/light (0–3 glass/week (0–30 g/week) for men and 0–2 (0–20 g/week) for women), moderate (4–21 (40–210 g/week) glass/week for men and 3–14 (30–140 g/week) for women) and heavy drinkers (>21 glass/week (>210 g/week) for men and >14 (>140 g/week) for women)^60^. We considered heavy drinking to be an unhealthy behaviour.

### Covariates

Sociodemographic measures: age, sex, education and monthly income/household were collected with a self-reported questionnaire. Body mass index (BMI, kg/m²) was measured at the examination.

Participants were considered diabetic if they reported that they had been declared as diabetic by a physician (or professional health worker) in the past, or if they were currently using anti-diabetic treatment (oral agents or injections), or if they were declared diabetic by the physician in the Health Screening examination, or if fasting blood glucose was ≥7 mmol/L at the HSC examination and non-diabetic otherwise.

Participants were considered as having hypercholesterolemia if any hypercholesterolemia was declared by the physician in the Health Screening examination or if fasting plasma total cholesterol at the Health Screening examination was ≥6.61 mmol/L and not having hypercholesterolemia otherwise.

Participants were considered as having hypertension if any hypertension has been reported by the physician in the Health Screening examination or if systolic blood pressure measured at the Health Screening examination was ≥140 mmHg or diastolic blood pressure was ≥90 mmHg and not having hypertension otherwise.

### Participants

**Figure 3:**
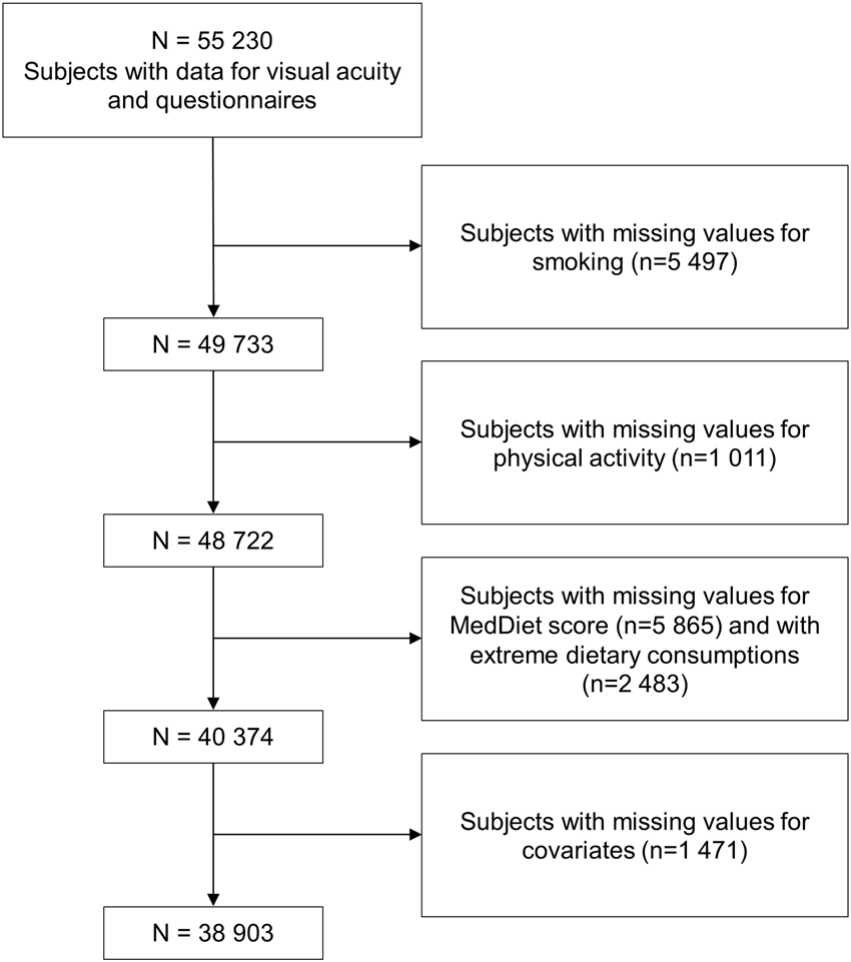
Flow chart showing the selection of subjects for our analyses.

Among participants included between February 2012 and January 2016, 55 230 had available data for visual acuity and questionnaires. Subjects who did not have available data for smoking (5 497), physical activity (1 011) and MedDiet score/alcohol (5 865) were excluded from our analyses (Fig. 3). We also excluded 2 483 subjects with extreme dietary consumptions (>99^th^ percentile of the distribution for at least one item of the MedDiet score). We thus included 38 903 (70.4%) participants with available data for visual acuity, MedDiet score, smoking, physical activity, sociodemographic and medical data.

### Statistical methods

Each characteristic was compared between subjects with and without visual impairment using mixed logistic regressions adjusted for age and sex with random intercept for health screening center.

We estimated the association between visual impairment and unhealthy behaviours using logistic mixed-effect models, with random intercept for health screening center. In a first step, models were adjusted for age (18–30, 30–40, 40–50, 50–60, 60–70 and ≥70 years), sex, education (≤primary school, secondary school, high school, ≤bachelor level and ≥master level or equivalent) and monthly income (<1500, 1500 to 2800, 2800 to 4200, ≥4200 euros/household) (Model 1). In a second step, models were further adjusted for diabetes, hypertension, hypercholesterolemia and BMI (<25, 25–30, ≥30 kg/m²) (Model 2).

We first performed separate models for each unhealthy behaviour using categorical variables and binary variables. Then, we examined the association between visual impairment and an unhealthy behaviours score, constructed as the number of unhealthy behaviours independently associated with visual impairment.

Characteristics of subjects according to the number of unhealthy behaviors were compared by using mixed multinomial logistic regressions adjusted for age and sex with random intercept for health screening center.

#### Sensitivity analyses

We evaluated whether associations between the number of unhealthy behaviours and visual impairment may differ using a different cut-off for diet quality. We used the median value of the MedDiet score (27 points) as an alternate cut-off and unhealthy behaviour was defined as very low/low diet quality.

To control for possible bias due to missing data, we imputed data for covariates and behaviours with missing data using a multiple imputations procedure. Five imputations were conducted taking the missing-at-random assumption and multivariate imputations by Chained Equations method^61^. Models were estimated for each imputation and were combined using Rubin’s rules with MIANALYSE procedure^62^.

All statistical analyses were performed with SAS 9.4 (SAS Institute) and p ≤ 0.05 was considered significant.

### Data availability

The datasets generated during and/or analysed during the current study are available from the CONSTANCES principal investigator (marie.zins@inserm.fr) provided that the procedures described in the CONSTANCES Charter (http://www.constances.fr/charter) are fulfilled.
